# PolyC-Binding Protein 1 Interacts with 5′-Untranslated Region of Enterovirus 71 RNA in Membrane-Associated Complex to Facilitate Viral Replication

**DOI:** 10.1371/journal.pone.0087491

**Published:** 2014-01-29

**Authors:** Zhen Luo, Xingchen Dong, Youxing Li, Qi Zhang, Cholho Kim, Yu Song, Lei Kang, Yingle Liu, Kailang Wu, Jianguo Wu

**Affiliations:** 1 State Key Laboratory of Virology and College of Life Sciences, Wuhan University, Wuhan, Hubei, China; 2 Chinese-French Liver Disease Research Institute, Zhongnan Hospital, Wuhan University, Wuhan, Hubei, China; University of Massachusetts Medical Center, United States of America

## Abstract

Enterovirus 71 (EV71) is one causative agent of hand, foot, and mouth disease (HFMD), which may lead to severe neurological disorders and mortality in children. EV71 genome is a positive single-stranded RNA containing a single open reading frame (ORF) flanked by 5′-untranslated region (5′UTR) and 3′UTR. The 5′UTR is fundamentally important for virus replication by interacting with cellular proteins. Here, we revealed that poly(C)-binding protein 1 (PCBP1) specifically binds to the 5′UTR of EV71. Detailed studies indicated that the RNA-binding K-homologous 1 (KH1) domain of PCBP1 is responsible for its binding to the stem-loop I and IV of EV71 5′UTR. Interestingly, we revealed that PCBP1 is distributed in the nucleus and cytoplasm of uninfected cells, but mainly localized in the cytoplasm of EV71-infected cells due to interaction and co-localization with the viral RNA. Furthermore, sub-cellular distribution analysis showed that PCBP1 is located in ER-derived membrane, in where virus replication occurred in the cytoplasm of EV71-infected cells, suggesting PCBP1 is recruited in a membrane-associated replication complex. In addition, we found that the binding of PCBP1 to 5′UTR resulted in enhancing EV71 viral protein expression and virus production so as to facilitate viral replication. Thus, we revealed a novel mechanism in which PCBP1 as a positive regulator involved in regulation of EV71 replication in the host specialized membrane-associated replication complex, which provides an insight into cellular factors involved in EV71 replication.

## Introduction

Enterovirus 71 (EV71), a member of the genus Enterovirus of Picornaviridae family, is the causative pathogen of hand, foot, and mouth disease (HFMD) in young children [1]. Acute EV71 infection can also cause severe neurological diseases and results in mortality in newborns [2], [3]. After its initial identification in the United States in 1969, EV71 outbreaks have been reported in Australia, Asia, and Europe [3]. Recent outbreaks of EV71 in China have affected millions and caused life-threatening complications in young children [4], [5]. EV71 is a non-enveloped virus with positive and single-stranded RNA of about 7400 nt that encodes a large polyprotein with a single open reading frame (ORF) flanked by 5′-untranslated region (5′UTR) and 3′UTR [6]. The polyprotein divides into three regions: P1 containing capsid proteins (VP1, VP2, VP3, and VP4), P2 and P3 containing non-structural proteins crucial to virus replication (2A, 2B, 2C, 3A, 3B, 3C, and 3D) [7].

The 5′UTR of EV71 RNA is about 745 nt and consists of two secondary structures: a cloverleaf structure involving in viral RNA replication and an internal ribosome entry site (IRES) directing initiation of translation [8]. During typical IRES-dependent translation in picornavirus, heterogeneous nuclear ribonucleoprotein A1 and K (hnRNP A1 and hnRNP K), and far upstream element-binding protein 1 and 2 (FUBP1 and FUBP2) interact with IRES of the viral 5′UTR to regulate initiation of translation of viral RNA [9], [10], [11], [12]. During viral genome replication, the cloverleaf structure in poliovirus (PV) RNA acts as a *cis*-acting element essential for negative-strand synthesis, which requires a membrane-associated replication complex of viral and cellular proteins along with a viral RNA template. Viral proteins 3CD and VPg along with cellular proteins poly(A)-binding protein (PABP) and poly(C)-binding protein (PCBP) interact with each other and with the ends of the viral RNA to form a circular ribonucleoprotein (RNP) complex [13], [14].

Human PCBP1, also called heterogeneous nuclear ribonucleoprotein E1 (hnRNP E1), is a protein of 38 kDa and one of four isoforms of the hnRNP E protein family. Similar to other members of hnRNP E family, PCBP1 has three RNA-binding domains called K-homologous 1, 2, and 3 (KH1, KH2, and KH3), of which KH1 and KH3 bind specifically to poly(C) homopolymers [15]. This protein can bind to the 3′-noncoding region (NCR) of cellular mRNAs and play a role in the stabilization or translational control of mRNAs [16], [17], [18]. It also interacts with the 5′UTR of poliovirus (PV) RNA to facilitate the viral RNA replication [19], [20]. In addition, PCBP1 plays a physiological role by mediating housekeeping degradation of mitochondrial antiviral signaling (MAVS) as a feedback inhibitor of antiviral immunity after viral infection [21]. PCBP1 can mediate interactions between iron or nucleic acids and proteins [22]. Although the effects of PCBP1 on translation and RNA replication of several viruses have been investigated, including PV, coxsackievirus (CAV), Kaposi's sarcoma-associated herpesvirus (KSHV), and porcine reproductive and respiratory syndrome virus (PRRSV) [15], [23], [24], [25], the mechanism involved in such regulation is largely unclear. In addition, the sub-cellular location of PCBP1 during EV71 replication is still unknown.

In this study, we at the first time investigated the effect of PCBP1 in control of EV71 replication and revealed the mechanism involved in such regulation. We showed that cellular PCBP1 can specifically interact with the 5′UTR of EV71 RNA. Detailed studies further revealed that PCBP1 binds to the stem-loops I and IV of EV71 5′UTR and the KH1 domain of PCBP1 is responsible for such binding.

All positive-strand RNA viruses, such as PV, CAV, and hepatitis C virus (HCV), rely on host intracellular membranes for replication. Upon infection, the viral RNA can be directly translated into protein by host machinery, replicated, and assembled on modified intracellular membranes [26], [27]. Here, we revealed that PCBP1 is distributed throughout the nucleus and cytoplasm in uninfected cells, but mainly distributed in the cytoplasm of EV71-infected cells through interaction and co-localization with EV71 RNA. Moreover, our results demonstrated that PCBP1 protein and EV71 RNA are located in the ER-derived membrane where virus replication occurred in the cytoplasm of EV71-infected cells, suggesting PCBP1 protein is recruited in viral replication complex associated membrane. Finally, we demonstrated that PCBP1 promote EV71 replication for the binding of PCBP1 to 5′UTR resulted in enhancing EV71 replication.

## Materials and Methods

### Cell lines, virus growth, and virus titration

Human embryonal rhabdomyosarcoma (RD), human neuroblastoma (SK-N-SH), and HeLa cells were cultured in modified Eagle's medium (MEM) (Invitrogen, Carlsbad, CA), supplemented with 10% fetal bovine serum (FBS), 100 U/ml penicillin and 100 µg/ml streptomycin sulfate at 37°C in a 5% CO2 incubator. All cell lines were obtained from China Center for Type Culture Collection (CCTCC, Wuhan University, Wuhan, China). Enterovirus 71 (EV71) virus strain (Xiangyang-Hubei-09) was isolated previously in our laboratory (GenBank accession no. JN230523.1). Cells were infected with EV71 at the indicated multiplicities of infection (MOI) and unbound virus was washed away 2 h later. Infected cells were then cultured in fresh medium with 2% FBS. Virus titration was performed using RD cells in 96-well plates and expressed as 50%-tissue culture infectious dose (TCID50) per unit volume. EV71 titers conformed to the Poisson model in that 1 TCID50/ml = 0.69 pfu/ml (plaque forming unit per milliliter), as described previously [28].

### Plasmids, small interfering RNAs, and antibodies

PCBP1-expressing plasmids were constructed as follows. DNA fragments of full-length and truncated PCBP1 gene (Genbank accession no. NM_006196.3) were amplified from RD cell cDNA by PCR using specific primers (Table 1), pCMV-PCBP1-fwd and pCMV-PCBP1-rev, KH_1_-fwd and KH_1_-rev, KH_1-2_-fwd and KH_1-2_-rev, KH_2-3_-fwd and KH_2-3_-rev, KH_3_-fwd and KH_3_-fwd, respectively. The PCR products were then inserted into the *Eco*RI and *Sal*I sites of plasmid pCMV-Tag-Flag2B (Invitrogen) to generate pCMV-Flag-PCBP1, pCMV-Flag-KH_1_ (1-288 bp), pCMV-Flag-KH_1-2_ (1-510 bp), pCMV-Flag-KH_2-3_ (289-1071 bp) and pCMV-Flag-KH_3_ (510–1071 bp), respectively.

**Table 1:** List of primers used in the study^a^.

Description	Orientation	Sequence (5′– 3′)
pCMV-PCBP1	Fwd	ATAGAATTCATGGATGCCGGTGTGACTGAAA
	Rev	AATGTCGACCTAGCTGCACCCCATGCCCTTCTCAG
KH_1_	Fwd	ATTGAATTCATGGATGCCGGTGTGACTG
	Rev	TTAGTCGACGGGCCTGCTGGCCGCGGTACTGTTGGTCAT
KH_1-2_	Fwd	ATTGAATTCATGGATGCCGGTGTGACTG
	Rev	TTAGTCGACGAGCGTCTCCAGCATGACCAGGCAA
KH_2-3_	Fwd	ATAGAATTCCCGGTCACCCTGAGGCT
	Rev	AATGTCGACCTAGCTGCACCCCATGCCCTTCTCAG
KH_3_	Fwd	ATTGAATTCTCCCAGTCTCCGCAAG
	Rev	AATGTCGACCTAGCTGCACCCCATGCCCTTCTCAG
5′UTR	Fwd	ATTAAGGTTTTAAACAGCTGTGGGTTGTCA
	Rev	AATTCTAGAGGTTTTGCTGTGTTGAGGGT
5′UTR (1–565)	Fwd	ATTAAGGTTTTAAAACAGCTGTGGGTTGTCA
	Rev	TTATCTAGAAGGAAACACGGACACC
5′UTR (1–450)	Fwd	ATTAAGGTTTTAAAACAGCTGTGGGTTGTCA
	Rev	TTATCTAGAGGAGGACTACTAACTA
5′UTR (100–450)	Fwd	TTCAAGCTTGTGAAACTTAGAAGCAGC
	Rev	TTATCTAGAGGAGGACTACTAACTA
5′UTR (1–230)	Fwd	ATTAAGGTTTTAAAACAGCTGTGGGTTGTCA
	Rev	TTATCTAGAGCCGGATAACGAACGT
5′UTR (1–180)	Fwd	ATTAAGGTTTTAAAACAGCTGTGGGTTGTCA
	Rev	TTATCTAGAGGAAACAGAAGTGCTTGATCA
5′UTR (1–100)	Fwd	ATTAAGGTTTTAAAACAGCTGTGGGTTGTCA
	Rev	TTATCTAGAGCTGCTTCTAAGTTTCAC
5′UTR(230–450)	Fwd	ATTAAGGTTACGTTCGTTATCCGGC
	Rev	TTATCTAGAGGAGGACTACTAACTA
5′UTR (100–230)	Fwd	TTCAAGCTTGTGAAACTTAGAAGCAGC
	Rev	TTATCTAGAGCCGGATAACGAACGT
5′UTR check	Fwd	ACAATTAAAGAGTTGTTACCATATAGCTATTGGATTGGCC
	Rev	CATGTTTTGCTGTGTTGAGGGTCAAGAT
RPS16	Fwd	GCGCGGTGAGGTTGTCTAGTC
	Rev	GAGTTTTGAGTCACGATGGGC
si-PCBP1	Fwd	CACCAUUCCAAAUAACUUAdTdT
	Rev	UAAGUUAUUUGGAAUGGUGdTdT
si-Control	Fwd	UUCUCCGAACGUGUCACGUdTdT
	Rev	ACGUGACACGUUCGGAGAAdTdT

a Restriction sites are underlined; Fwd, forward; Rev, reverse; 5′UTR check, RT-PCR analysis of 5′UTR in EV71 genome; RPS16, RT-PCR analysis of ribosomal protein S16; siPCBP1 Fwd, sense sequence of small interfering against PCBP1; siPCBP1 Rev, anti-sense sequence of small interfering against PCBP1.

The full-length and truncated 5′UTR of EV71 RNA were amplified from EV71 cDNA using specific primers (Table 1), 5′UTR-fwd and 5′UTR-rev, 5′UTR (1–565)-fwd and 5′UTR (1–565)-rev, 5′UTR (1–450)-fwd and 5′UTR (1–450)-rev, 5′UTR (100–450)-fwd and 5′UTR (100–450)-rev, 5′UTR (1–230)-fwd and 5′UTR (1–230)-rev, 5′UTR (230–450)-fwd and 5′UTR (200–450)-rev, 5′UTR (1–180)-fwd and 5′UTR (1–180)-rev, 5′UTR (1–100)-fwd and 5′UTR (1–100)-rev, 5′UTR (100–230)-fwd and 5′UTR (100–230)-rev, respectively. The PCR products were then inserted downstream of the T7 promoter at the *Hind*III and *Xba*I sites of pcDNA3.0 (Invitrogen) to generate pcDNA3.0-5′UTR and deletions pcDNA3.0-5′UTR (1–565), (1–450); (100–450), (1–230), (1–180), and (100–230), respectively. Primers used in the study are list in Table 1 and the restriction sites are underlined. The sequences and orientations of all cloned cDNAs were confirmed by DNA sequencing analysis.

The siRNA specific to PCBP1 (siPCBP1) and the control siRNA (siCtrl) were synthesized by RiboBio (Guangzhou, China) according to the sequences of siPCBP1-fwd and siPCBP1-rev or siCtrl-fwd and siCtrl-rev (Table 1), respectively.

The primary antibodies used in this study include mouse anti-hnRNP E1 monoclonal antibody, mouse anti-β-actin monoclonal antibody, normal mouse IgG (Santa Cruz Biotechnology, Santa Cruz, CA, USA), rat anti-Flag monoclonal antibody (BioLegend, San Diego, CA, USA), rabbit anti-EV71-VP1 polyclonal antibody (Abnova, Taipei city, Taiwan), mouse anti-dsRNA (J2) monoclonal antibody (Scicons, Hungary), and rabbit anti-calnexin polyclonal antibody (ProteinTech Group, Chicago, IL, USA). The rabbit anti-EV71 2C polyclonal antibody was a gift from Professor Hanzhong Wang of Wuhan Institute of Virology, Chinese Academy of Sciences, Wuhan, China. The rabbit anti-EV71 3C polyclonal antibody was raised against residues 76–88 of 3C protein (Abgent, Suzhou, China). The purified recombinant PCBP1 and PCBP2 proteins were purchased from ProteinTech Group.

### Co-immunoprecipitation and RT-PCR

RD cells were infected with EV71 at an MOI of 40 and harvested at 6 h post-infection (p.i.). Lysates were pre-cleared by incubation on ice for 1 h with protein A/G (Santa Cruz Biotechnology) to bind non-specific antibodies. Non-specific complexes were pelleted by centrifugation at 10,000×*g* at 4°C for 10 min. The supernatants were removed and subjected to co-immunoprecipitation assays. 100 µl of pretreated lysate was diluted with 450 µl lysis buffer, and 20 µl of hnRNP E1 antibody was added. After incubation on ice for 2 h, 100 µl of pre-wash protein A/G (v/v%, 50% in PBS) was added and samples incubated on ice for 1 h. Complexes were pelleted by centrifugation at 1,000×*g* at 4°C for 5 min and washed five times with lysis buffer. Each pellet (or 100 µl of pre-cleared lysate for total RNA extraction) was resuspended in 400 µl of proteinase K buffer (100 mM Tris-HCl, pH 7.5, 12.5 mM EDTA, 150 mM NaCl, 1% SDS) and incubated with 100 µg of predigested proteinase K for 30 min at 37°C. RNA was phenol-chloroform extracted, precipitated in isopropanol at −20°C for 30 min, washed in 70% ethanol, dried and eluted in 20 µl DEPC H_2_O. Reverse-transcription PCR was performed with M-MLV Reverse Transcriptase (Promega, Madison, WI) to obtain cDNA, and specific DNA fragments were amplified using primers specific for EV71 5′UTR or ribosomal protein S16 RNA (Table 1).

### In vitro transcription and biotinylated RNA pull-down assays

Plasmids of pcDNA3.0 ligated with full length EV71 5′UTR and six truncated forms of EV71 5′UTR were linearized with *Xba*I, purified by LiAc, ethanol precipitation, and dissolved in 0.1×Tris-EDTA (TE) buffer. A total of 2 µg of restricted plasmid DNA was *in vitro* transcribed into RNA using the MEGAscript™ T7 kit (Ambion, Austin, TX, USA) and purified with a MEGA clear kit (Ambion) according to the manufacturer's protocol. The RNA concentration and integrity were determined using BioSpec-nano (Shimadzu Biotech, Kyoto, Japan). For RNA labeling, RNA fragments were ligated to Biotin-16-UTP (Roche) at the 3′ end with T4 RNA ligase (Promega) according to the manufacturer's instructions. For the biotinylated RNA binding assay, a reaction mixture containing 200 µg of cell extracts and 5 µg of biotinylated RNA was prepared. The mixture with a final volume of 100 µl was incubated in RNA mobility-shift buffer (5 mM HEPES pH 7.1, 40 mM KCl, 1 U RNasin, and 0.25 mg/ml heparin) for 15 min at 37°C, and then added to 400 µl of Streptavidin MagneSphere Paramagnetic Particles (Promega) and allowed to bind for 10 min at room temperature. The RNA-protein complexes were washed five times with RNA mobility-shift buffer without heparin. After the last wash, the complexes were dissolved in 30 µl of 2× SDS-PAGE sample buffer, boiled and subjected to 10–12% SDS-PAGE. Bound proteins were subjected to Western blotting using specific antibodies.

### Native gel mobility shift assay

EV71 5′-UTR RNA was denatured at 95°C for 3 min and cooled to 25°C for 10 min. The RNA and purified recombinant PCBP1 protein (rPCBP1) were then incubated together in binding buffer (20 mM Tris-Cl, pH 7.5, 5 mM MgCl_2_, 50 mM KCl, 1 mM DTT, 10% glycerol, and 2 U of RNasin) at 37°C for 20 min. The samples were loaded onto a native 8% PAGE and transferred to nitrocellulose membranes (Amersham, Piscataway, NY, USA). The membranes were then blocked with 10% fat-free powdered milk in PBST for 30 min, incubated with HRP-Streptavidin (Cell Signaling Technology, Beverly, MA, USA) for 1 h, washed, incubated with HRP substrate luminol reagent (Minipore, Billerica, MA, USA), and analyzed using a Luminescent Image Analyzer (Fujifilm LAS-4000, Tokyo, Japan).

### Immunofluorescence microscopy

RD cells were seeded on 20-mm cover slips to 90% confluence and infected with EV71 at an MOI of 5. At 6 or 12 h p.i., culture medium was removed and cells were washed three times with PBS and fixed with 3.7% formaldehyde for 20 min at room temperature. Cells were then washed with PBS and permeabilized using 0.4% Triton X-100 for 5 min at room temperature. After another PBS wash, cells were blocked in PBS containing 5% BSA for 1 h at 37°C, incubated with antibodies for 3 h at 37°C and washed three times with PBS. The samples were incubated with FITC-conjugated goat anti-mouse immunoglobulin G (IgG) and Cy3-conjugated goat anti-rabbit IgG (ProteinTech Group) for 45 min at room temperature. For live-cell ER labeling, 1 µM ER-Tracker™ Blue (Invitrogen) stained cells for 15 min at 37°C. To stain nuclei, 1 µg/ml DAPI (Roche) methanol solution was added and samples incubated for 15 min at room temperature. After washing with PBS, samples were visualized by confocal laser-scanning microscopy (Fluoview FV1000; Olympus, Tokyo, Japan).

### Membrane flotation and detergent solubilization assays

The assay was performed as previous described [29]. Briefly, cells were seeded in two 100-mm plates, uninfected or infected with EV71 (MOI = 5) for 8 h, lysed in 1 ml of hypotonic buffer (10 mM Tris-HCl, pH 7.5, 5 mM MgCl2, 10 mM KCl), and passed through a 25-guage needle at least 20 times. The supernatants were collected after removing nuclei and unbroken cells by centrifugation at 1,000×g at 4°C for 5 min, and then untreated or treated with 1% Triton X-100 for 1 h at 4°C. Cell lysates were mixed with 3 ml of 72% sucrose in low-salt buffer (LSB; 50 mM Tris-HCl, pH 7.5, 5 mM MgCl_2_, 25 mM KCl) and overlaid with 4 ml of 55% sucrose, followed by 1.5 ml of 10% sucrose in LSB. The sucrose gradient centrifugation was performed in a Beckman SW41 Ti rotor at 38,000 rpm for 14 h at 4°C. From the top of the gradient, 1-ml fractions were taken and each one was added 1.7 ml of LSB to dilute sucrose. The fractions were concentrated by being passed through an Amicon Ultra 100 k filter (Millopore) and subjected to Western blotting using specific antibodies.

### Preparation of cell extracts

RD, SK-N-SH, and HeLa cells were cultured in MEM supplemented with 10% FBS. Cells were pelleted, washed twice with cold PBS, and resuspended in RIPA buffer (50 mM Tris, pH 7.5, 150 mM NaCl, 1% Triton X-100, 1% sodium deoxycholate, 0.1% SDS, 1 mM sodium formate, 1 mM phenylmethylsulfonyl fluoride, and 10% protease inhibitor cocktail) on ice for 30 min. Lysates were centrifuged at 10,000×*g* for 10 min. Protein concentrations were determined using the Bradford assay kit (Bio-Rad, Hercules, CA). For nuclear extraction, cells were collected, washed twice with ice-cold PBS, and scraped into 1 ml cold PBS. Cells were pelleted by centrifugation for 15 s and incubated in two packed cell volumes of buffer A (10 mM HEPES, pH 8.0, 0.5% Nonidet P-40, 1.5 mM MgCl_2_, 10 mM KCl, 0.5 mM DTT, and 200 mM sucrose) for 5 min at 4°C with flipping of the tube. The crude nuclei were collected by centrifugation for 30 s and pellets were rinsed with buffer A, resuspended in one packed cell volume of buffer B (20 mM HEPES, pH 7.9, 1.5 mM MgCl_2_, 420 mM NaCl, 0.2 mM EDTA, and 1.0 mM DTT), and incubated on a shaking platform for 30 min at 4°C. Nuclei were centrifuged for 5 min and supernatants were diluted 1∶1 with buffer C (20 mM HEPES, pH 7.9, 100 mM KCl, 0.2 mM EDTA, 20% glycerol, and 1.0 mM DTT). Cocktail protease inhibitor (Roche, Mannheim, Germany) tablets were added to each buffer. Nuclear extracts were snap-frozen in liquid nitrogen and stored at −70°C until use.

### Western blot analysis

After preparation of cell extracts, the lysates (100 µg) were electrophoresed on a 12% SDS-PAGE gel and transferred to a nitrocellulose membrane (Amersham, Piscataway, NY). Nonspecific sites were blocked with 5% nonfat dried milk and then incubated with the primary antibody. Blots were analyzed using a Luminescent Image Analyzer (Fujifilm LAS-4000).

### Statistics

All experiments were reproducible and each set was repeated at least three times. Parallel samples were analyzed for a normal distribution using the Kolmogorov-Smimov test. Abnormal values were eliminated according to a follow-up Grubbs test. Levene's test for equality of variances was performed, which provided information to determine the equality of means using Student's *t*-test. Means are expressed as a histogram with error bars representing ± SD, and *p*<0.05 was considered to indicate statistical significance.

## Results

### PCBP1 specifically binds to the 5′UTR of EV71 RNA

**Figure 1:**
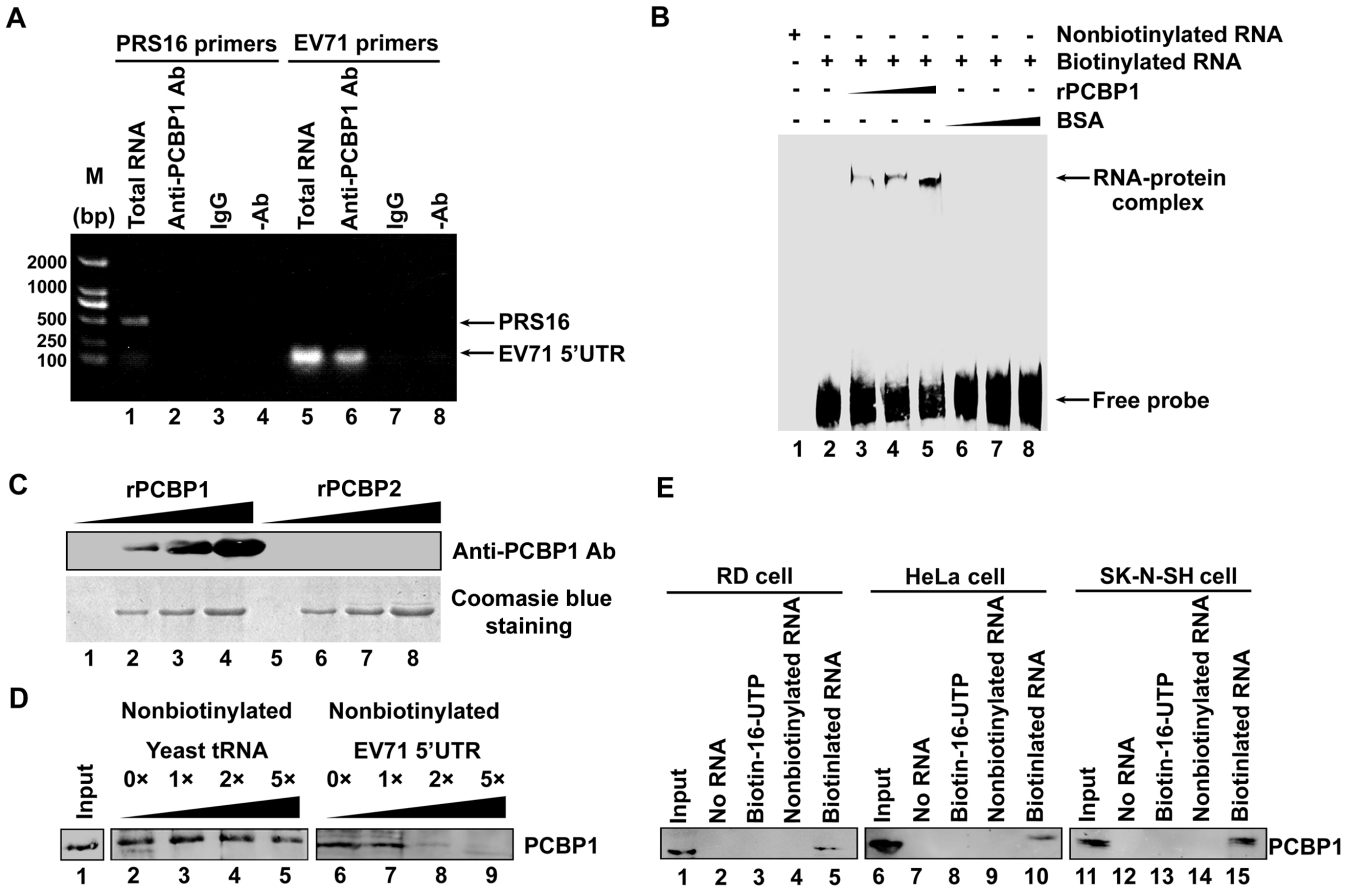
Interaction between PCBP1 protein and the 5′UTR of EV71 RNA. (A) Co-immunoprecipitation (Co-IP) and RT-PCR assay. RD cells were infected with EV71 at an MOI of 40 for 6 h and then cell extracts were prepared. For Co-IP assay, extract of EV71-infected cells was incubated with mouse anti-PCBP1 antibody (lanes 2 and 6), normal mouse IgG (lanes 3 and 7), or without antibody (-Ab) (lanes 4 and 8). Following washing and dissociation, the RNA extract was prepared and subjected to RT-PCR analysis with primers specific for the ribosomal protein S16 (PRS16) RNA (lanes 1–4) or for EV71 5′UTR RNA (lanes 5–8). Total RNA from cell extracts without Co-IP was evaluated by RT-PCR as a control (lanes 1 and 5). RT-PCR products were separated and detected by agarose gel electrophoresis and the expected band is indicated by an arrow. (B) Electrophoretic mobility shift assay (EMSA). Plasmid carrying EV71 5′UTR DNA was linearized and *in vitro* transcribed into RNA, which was then labeled with biotin-16-UTP at the 3′ end. Labeled RNA (0.5 µg) was incubated with recombinant PCBP1 (rPCBP1) at 0, 0.1, 0.15 and 0.2 µg (lanes 2 to 5) or BSA at 0.1, 0.15, and 0.2 µg (lanes 6 to 8), respectively. The RNA-protein complex (indicated by an arrow) was separated from free probe RNA and examined by EMSA. No-labeled RNA was in lane 1 as a control. (C) The specificity of the PCBP1 antibody. Purified rPCBP1 protein (lanes 1 to 4) and rPCBP2 protein (lanes 5 to 8) were subjected to SDS-PAGE at 0, 2, 4 and 8 µg, respectively, stained by Coomasie blue and then detected by specific PCBP1 antibody. (D) Biotinylated RNA/protein pull-down assays. For RNA/protein pull-down assay and for competition assay, cell extracts were mixed with biotinylated EV71 5′UTR RNA (lanes 2–9) along with different concentrations of nonbiotinylated yeast tRNA (lanes 2–5) or nonbiotinylated EV71 5′UTR RNA (lanes 6–9), respectively. After pull-down assay, the bound proteins were boiled and subjected to 12% SDS-PAGE and analyzed by western blotting with a mouse anti-PCBP1 antibody. The input contained 200 µg cell lysate (lane 1). (E) Extracts of RD cells (lanes 1–5), HeLa cells (lanes 6–10), and SK-N-SH cells (lanes 11–15) were prepared and then incubated without RNA (lanes 2, 7, and 12), with biotin-16-UTP (lanes 3, 8, and 13), non-biotinylated EV71 5′UTR (lanes 4, 9, and 14), or biotinylated EV71 5′UTR (lanes 5, 10, and 15). After pull-down assay, the bound proteins were boiled, eluted, and subjected to 12% SDS-PAGE and PCBP1 protein was detected by western blot with a mouse anti-PCBP1 antibody. The inputs were cell extracts of RD (lane 1), HeLa (lane 6), and SK-N-SH (lane 11).

To evaluate the role of PCBP1 in the replication of EV71, we first investigated the binding ability of PCBP1 protein to the 5′UTR of EV71 RNA. RD cells were infected with EV71, cell extracts were prepared, and RNA was determined by RT-PCR. The association of PCBP1 with EV71 5′UTR was then determined by co-immunoprecipitation (Co-IP) assay using mouse antibody to PCBP1 and with or without mouse IgG, respectively, followed by RT-PCR. Results revealed that ribosomal protein S16 (RPS16) RNA and EV71 5′UTR RNA were detected in the total RNA extracts (Fig. 1A, lanes 1 and 5). However, RPS16 RNA was not detected after Co-IP (Fig. 1A, lanes 2, 3, and 4), while EV71 5′UTR RNA was detected after Co-IP in the presence of anti-PCBP1 antibody (Fig. 1A, lane 6), but not detected in the presence of mouse IgG (Fig. 1A, lane 7) and absence of the antibody (Fig. 1A, lane 8). These results suggested that cellular PCBP1 protein interacts with the 5′UTR of EV71 RNA in EV71-infected cells.

The ability of PCBP1 protein binds to the 5′UTR of EV71 was further determined by electrophoretic mobility shift assay (EMSA). Labeled EV71 5′-UTR RNA (nt 1–745) was incubated with purified PCBP1 protein (0.1, 0.15, and 0.2 µg) or BSA (0.1, 0.15, and 0.2 µg) at different concentrations. EMSA results showed that a RNA-protein complex is formed in the presence of PCBP1 protein and the intensity of the complex increased as the concentration of PCBP1 protein increased (Fig. 1B, lanes 3-5). However, RNA-protein complex was not detected in the presence of BSA (Fig. 1B, lanes 6–8). The results suggested that PCBP1 specifically binds to EV71 5′UTR. Since PCBP1 and PCBP2 are similar proteins, we evaluated the specificity of PCBP1 antibody to discriminate them. The purified recombinant PCBP1 and PCBP2 were subjected to 10% SDS PAGE followed by Coomasie blue staining (Fig. 1C, lower panel), and then immunoblotting by using specific anti-PCBP1 antibody. It showed that PCBP1 antibody specifically detects PCBP1 protein (Fig. 1C, lanes 1–4) but not PCBP2 protein (Fig. 1C, lanes 5–8) at different concentrations.

Specific interaction between PCBP1 and EV71 5′UTR was further evaluated by biotinylated RNA pull-down assays. Plasmid carrying EV71 5′UTR DNA was linearized, *in vitro* transcribed into RNA, which was then labeled with biotin-16-UTP. For the pull-down assay, cell extracts were mixed with biotinylated EV71 5′UTR RNA along with different concentrations of non-biotinylated yeast tRNA and non-biotinylated EV71 5′UTR RNA, respectively. PCBP1 protein was determined by western blot using a mouse anti-PCBP1 antibody. Results showed that PCBP1 was detected in the cell lysate (input) (Fig. 1D, line 1) and pull-downed by biotinylated EV71 5′UTR (Fig. 1D, lanes 2–9). In addition, the level of PCBP1 protein was not affected by non-biotinylated yeast tRNA (Fig. 1D, lanes 2–5), but reduced in the presence of non-biotinylated EV71 5′UTR RNA in a dose-dependent manner (Fig. 1D, lanes 6–9). These results further confirmed that PCBP1 specifically binds to EV71 5′UTR RNA.

Since PCBP1 is widely expressed in human and mouse cells [30], we further determined whether PCBP1 can bind to EV71 of 5′UTR RNA in different cell types. RD, HeLa, and SK-N-SH cells extracts were prepared and subjected to RNA affinity-purification assays. Cell extracts were incubated without RNA or with biotin-16-UTP, nonbiotinylated EV71 5′UTR, and biotinylated EV71 5′UTR, respectively. Results showed that PCBP1 protein was detected in all cell extracts (inputs) prepared from RD, HeLa, and SK-N-SH cells (Fig. 1E, lanes 1, 6, and 11). After pull-down assay, PCBP1 protein was only detected in the presence of biotinylated EV71 5′UTR (Fig. 1E, lanes 5, 10, and 15), but not detected in the absence of RNA (Fig. 1E, lanes 2, 7, and 12), in the presence of biotinylated 16-UTP (Fig. 1E, lanes 3, 8, and 13), or in the presence of nonbiotinylated EV71 5′UTR (Fig. 1E, lanes 4, 9, and 14). The results demonstrated that PCBP1 can specifically bind to EV71 5′UTR RNA in various cell types.

### PCBP1 binds to the stem-loop I and IV within the 5′UTR of EV71

**Figure 2:**
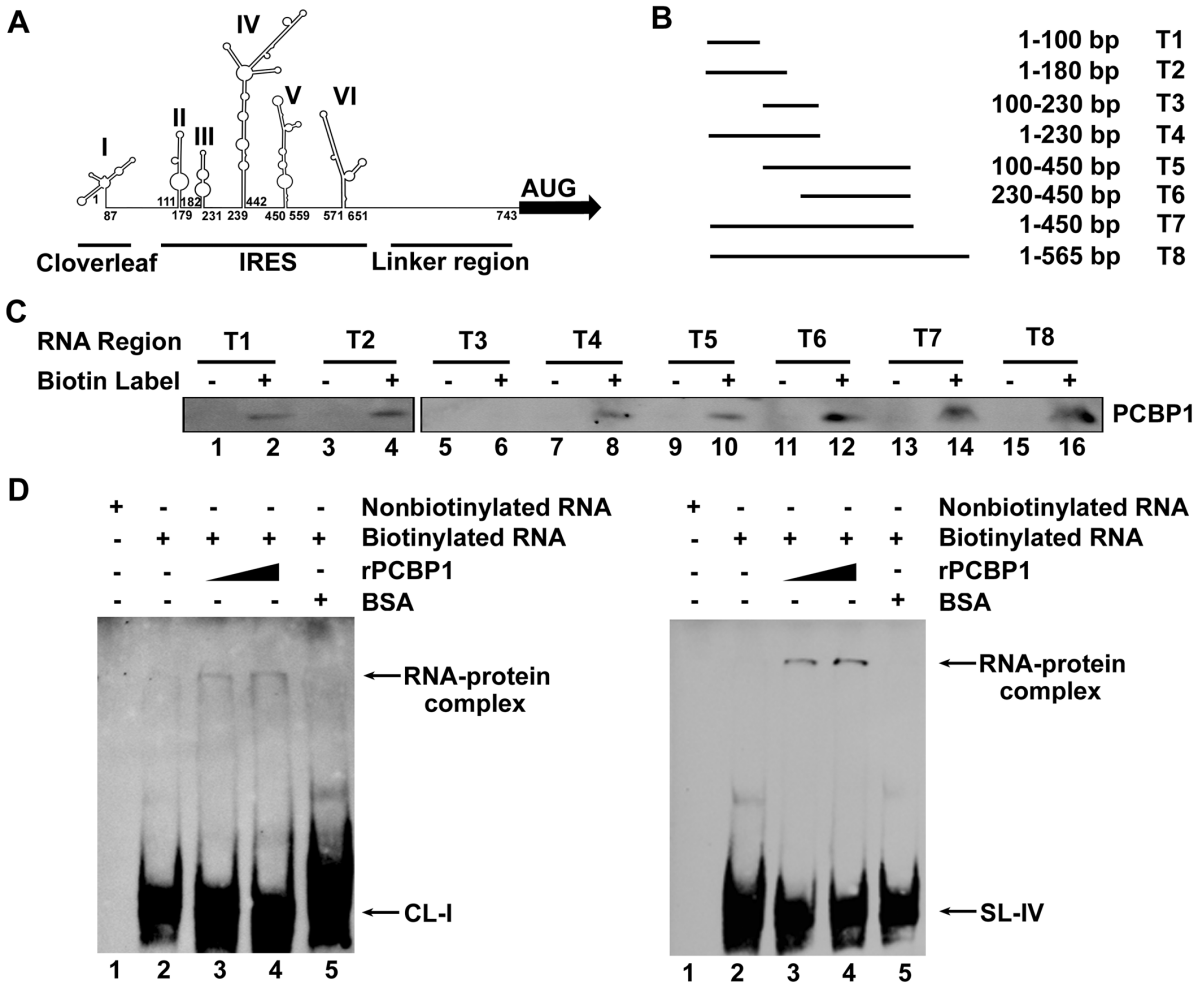
Determination of the sequences in 5′UTR of EV71 RNA required for the binding of PCBP1. (A) The secondary structure of the full-length 5′UTR of EV71 RNA was predicted using the M-FOLD software (http://mfold.rna.albany.edu). In this predicted secondary structure, six domains (or stem-loops) were shown with first and last nucleotides numbered: domain I (nt 1 to 87), II (nt 111 to 179), III (nt 182 to 231), IV (nt 239 to 442), V (nt 450 to 559), and VI (nt 571 to 651). The three functional regions, cloverleaf, IRES, and linker, were underlined based on previous report [11]. (B) A series of plasmids carrying different deletions in the six stem-loops of EV71 5′UTR were generated: pcDNA3.0-5′UTR (T1, 1–100), (T2, 1–180), (T3, 100–230), (T4, 1–230), (T5, 100–450), (T6, 230–450), (T7, 1–450), and (T8, 1–565). (C) The plasmids were linearized and transcribed into RNA *in vitro*, which were then labeled with biotin-16-UTP. For the pull-down assay, cell extracts were mixed with these biotinylated EV71 5′UTR RNAs or nonbiotinylated RNAs as control (lanes 1, 3, 5, 7, 9, 11, 13, and 15). The complex was dissolved and PCBP1 protein was determined by western blot using a mouse anti-PCBP1 antibody (lanes 2, 4, 6, 8, 10, 12, 14, and 16). (D) EMSA assay for domain I (CL-I) and IV (SL-IV). Labeled RNA (CL-1 or SL-IV) (0.5 µg) was incubated with rPCBP1 at 0, 0.1, and 0.2 µg (lanes 2 to 4) or BSA at 0.2 µg (lane 5), respectively. No-labeled RNA was used as a control (lane 1). The RNA-protein complex was separated from free probe RNA and examined by an EMSA. The bound RNA is indicated by an arrow.

The 5′UTR of enterovirus genome contains six domains (or stem-loops, SL), stem-loop I is a cloverleaf (CL) structure that is important for virus replication [31], while stem-loop II–VI encompass an internal ribosome entry site (IRES), which directs translation of the viral RNA through internal ribosome binding [32]. The 5′UTR of EV71 RNA comprises 745 nt and forms a secondary structure divided into three functional areas: the cloverleaf, IRES, and linker regions [33]. In this study, we predicted the secondary structures of the 5′UTR of EV71 using M-FOLD (http://mfold.rna.albany.edu). In this predicted secondary structures, six domains were shown, domain I (nt 1 to 87), II (nt 111 to 179), III (nt 182 to 231), IV (nt 239 to 442), V (nt 450 to 559), and VI (nt 571 to 651) (Fig. 2A). The three functional regions, cloverleaf, IRES and linker were underlined based on previous report [11].

To determine which domains are responsible for PCBP1 binding, a series of plasmids carrying different deletions in the six stem-loops of EV71 5′UTR were generated (Fig. 2B): pcDNA3.0-5′UTR-T1, T2, T3, T4, T5, T6, T7, and T8. These plasmids were then linearized, transcribed into RNA *in vitro*, which were then labeled with biotin-16-UTP. For the pull-down assay, cell extracts were mixed with biotinylated or nonbiotinylated EV71 5′UTR RNAs and PCBP1 protein was determined by western blot. Results showed that PCBP1 was detected in the presence of 5′UTR-T1 RNA, 5′UTR-T2 RNA, 5′UTR-T4 RNA, 5′UTR-T5 RNA, 5′UTR-T6 RNA, 5′UTR-T7 RNA, and 5′UTR-T8 RNA (Fig. 2C, lane 2, 4, 8, 10, 12, 14, and 16), but not detected in the presence of 5′UTR-T3 RNA (Fig. 2C, lane 6). These results suggested that EV71 5′UTR stem-loop I (nt 1–100) and stem-loop IV (nt 230–450) are required for the binding of PCBP1 protein.

The ability of PCBP1 binds to stem-loop I (CL-I) or stem-loop IV (SL-IV) of EV71 5′UTR was further evaluated by EMSA assay. Labeled CL-I and SL-IV were incubated with purified PCBP1 protein at different concentrations or BSA as a control, respectively. Results from CL-I and PCBP1 binding assay showed that a RNA-protein complex is formed in the presence of PCBP1 protein and the intensity of the complex increased as the concentration of PCBP1 increased (Fig. 2D, lanes 2–4, left panel), but RNA-protein complex is not detected in the presence of BSA (Fig. 2D, lane 5, left panel). Similarly, results from CL-I and PCBP1 binding assay revealed that a RNA-protein complex is formed in the presence of PCBP1 protein and the intensity of the complex increased as the concentration of PCBP1 increased (Fig. 2D, lanes 2–4, right panel), but RNA-protein complex is not detected in the presence of BSA (Fig. 2D, lane 5, right panel). Thus, our results demonstrated that stem-loop I and IV of EV71 5′UTR are required for the binding of PCBP1.

### The KH1 domain of PCBP1 is responsible for the binding of PCBP1 to EV71 5′UTR

**Figure 3:**
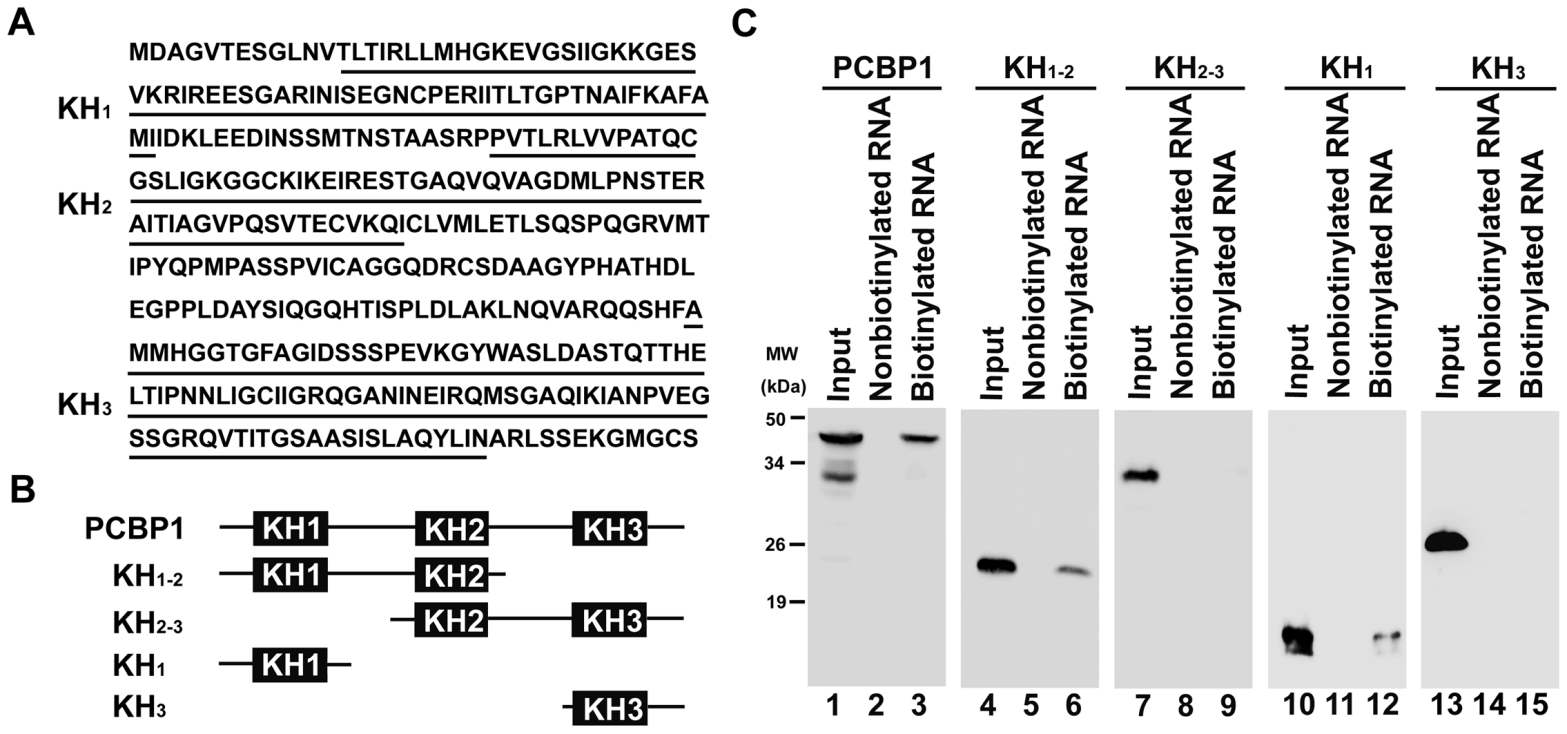
Analysis of the function domain of PCBP1 protein required for its binding to the 5′UTR of EV71 RNA. (A) The KH domains of full-length PCBP1 protein were predicted by PROSITE tools (http://prosite.expasy.org/). The amino acids of the three KH domains, KH1, KH2, and KH3, were underlined. (B) Based on the sequences of PCBP1 protein and its predicted domains, five plasmids expressing the full-length PCBP1 or truncated PCBP1 proteins were constructed, in which the KH3 was deleted (KH_1-2_), the KH1 was deleted (KH_2-3_), both KH2 and KH3 were deleted (KH_1_), or both KH1 and KH2 were deleted (KH_3_), respectively. (C) For the RNA-protein binding assay, HeLa cells were transfected with plasmids coding the Flag-tagged full-length PCBP1 (lanes 1-3) or the Flag-tagged truncated proteins, PCBP1-KH_1-2_ (lanes 4–6), PCBP1-KH_2-3_ (lanes 7–9), PCBP1-KH_1_ (lanes 10–12), and PCBP1-KH_3_ (lanes 13–15), respectively. For the pull-down assay, cell extracts were prepared and mixed with nonbiotinylated EV71 5′UTR RNA (lanes 2, 5, 8, 11, and 14) or biotinylated EV71 5′UTR RNA (lanes 3, 6, 9, 12, and 15), respectively. The complexes were boiled and dissolved and PCBP1 protein was determined by western blot analysis using anti-Flag antibody (lanes 2, 3, 5, 6, 8, 9, 11, 12, 14, and 15). The inputs were cell extracts without pull-down assay (lanes 1, 4, 7, 10, and 13).

PCBP1 contains three RNA-binding KH domains referred to KH1, KH2, and KH3. The KH1 and KH3 are thought to specifically bind to poly(C) homopolymers [15]. Based on the sequences of PCBP1 protein and its predicted domains (Fig. 3A), we constructed five plasmids expressing the full-length PCBP1 protein and four truncated PCBP1 proteins, KH_1-2_, KH_2-3_, KH_1_ and KH_3_, respectively (Fig. 3B). For the RNA-protein binding assay, cells were transfected with these plasmids and cell extracts were prepared and mixed with nonbiotinylated or biotinylated EV71 5′UTR RNA, respectively. Results showed that all five PCBP1 proteins were expressed in transfected cells and detected in the inputs (Fig. 3C, lanes 1, 4, 7, 10, and 13). We also revealed that PCBP1, KH_1-2_, and KH_1_ proteins were detected in the presence of biotinylated EV71 5′UTR RNA (Fig. 3C, lanes 3, 6, and 12), but KH_2-3_ and KH_3_ protein was not detected in the presence of biotinylated EV71 5′UTR RNA (Fig. 3C, lanes 9 and 15). As negative controls, PCBP1, KH_1-2_, KH_2-3_, KH_1_, and KH_3_ proteins were not detected in the presence of nonbiotinylated EV71 5′UTR RNA (Fig. 3C, lanes 2, 5, 8, 11, and 14). Thus, our results indicated that KH1 domain of PCBP1 is responsible for the binding of PCBP1 to the 5′UTR of EV71 RNA.

### PCBP1 is co-localized with EV71 RNA and mainly distributed in cytoplasm in infected cells

**Figure 4:**
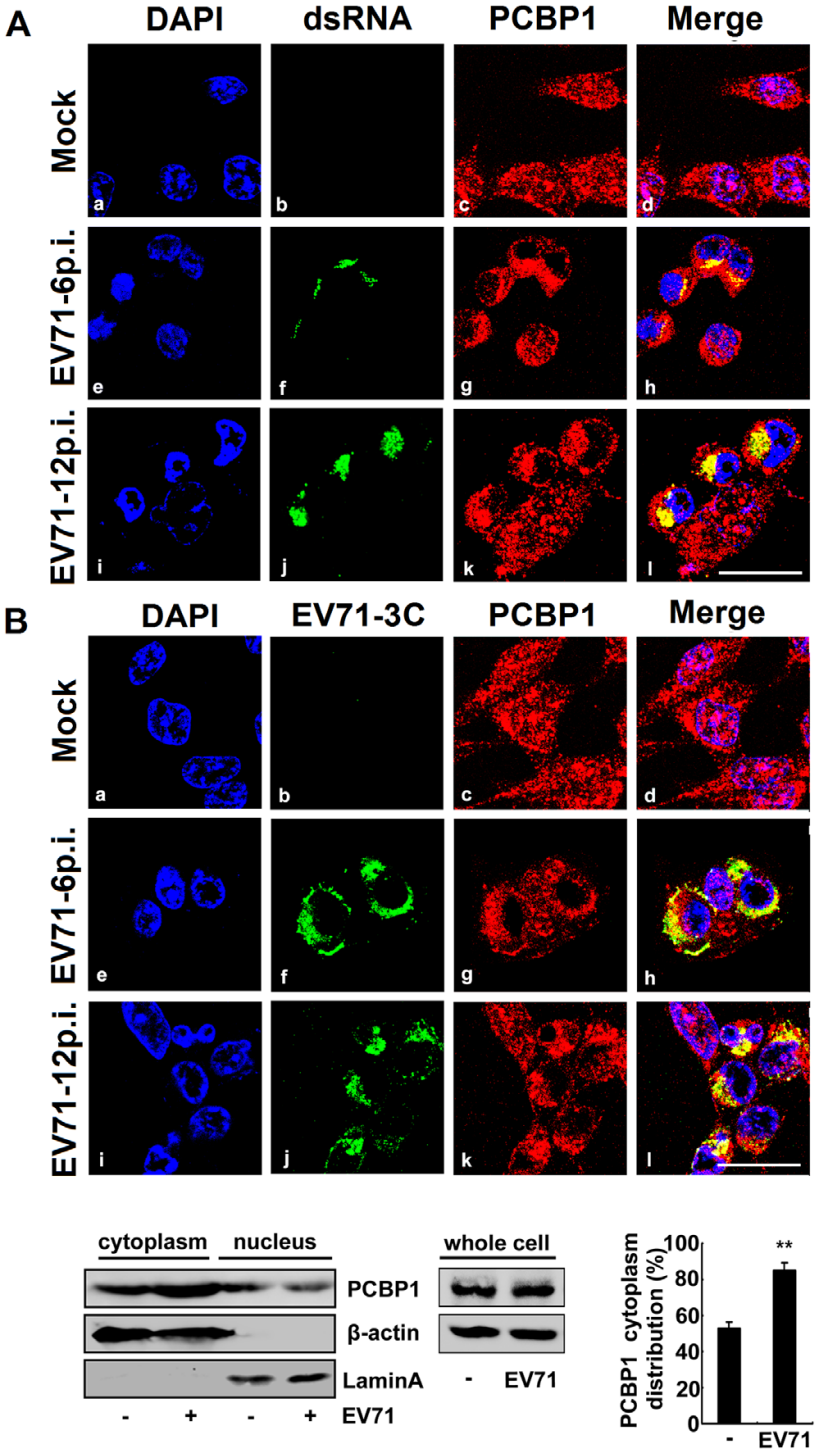
Subcellular distribution of PCBP1 protein during EV71 infection. (A) RD cells were mock-infected or infected with EV71 at an MOI of 5 for 6 and 12 h. Cells were probed with rabbit anti-PCBP1 antibody and mouse anti-dsRNA antibody, stained with DAPI, examined by confocal microscopy (Fluoview FV1000; Olympus). PCBP1 (red), dsRNA (green), DAPI (blue). Bar = 20 µm. (B) RD cells were mock-infected or infected with EV71 at an MOI of 5 for 6 and 12 h. Cells were probed with mouse anti-PCBP1 antibody and rabbit anti-EV71 3C antibody, stained with DAPI and examined by confocal microscopy. PCBP1 (red), EV71 3C (green), DAPI (blue). Bar = 10 µm. (C) RD cells were infected with or without EV71 at an MOI of 5 for 12 h. Whole cell lysates, cytoplasm, and nucleus extractions were prepared from mock-infected and EV71-infected cells, respectively. Endogenous proteins were detected by western blot analysis using antibody to PCBP1 or β-actin. The blot is a representative of three independent experiments with similar results. The bar graph showed change of PCBP1 distribution based on three independent experiments with similar results (**, *p*<0.01).

PCBP1 is normally distributed throughout the cells, including the nucleus and cytoplasm [34]. All positive-strand RNA viruses, such as poliovirus and HCV, replicate and translate in the cytoplasm of host cells [35]. Here, we evaluated sub-cellular distribution of PCBP1 and EV71 RNA during EV71 infection. RD cells were infected with or without EV71 and then probed with anti-PCBP1 and anti-dsRNA antibodies, stained with DAPI, and examined by confocal microscopy. In mock-infected cells, dsRNA was not detected as expected (Fig. 4Ab), while PCBP1 protein was detected and distributed throughout the nucleus and cytoplasm (Fig. 4Ac and 4Ad). In EV71-infected cells, EV71 replication (indicated as dsRNA) was occurred in the cytoplasm at 6 h and 12 h post-infection (Fig. 4Af and 4Aj), while PCBP1 protein was mainly distributed in the cytoplasm (Fig. 4Ag and 4Ak). More interestingly, a proportion of PCBP1 was co-localized with dsRNA in the cytoplasm of EV71-infected cells (Fig. 4Ah and 4Al) due to binding to the viral RNA.

In addition, we investigated sub-cellular distributions of PCBP1 protein and EV71 3C protein during EV71 infection under similar conditions. Results showed that in mock-infected cells, EV71 3C protein was not detected (Fig. 4Bb) as expected, while PCBP1 protein was detected and distributed throughout the nucleus and cytoplasm (Fig. 4Bc and 4Bd). In EV71-infected cells, EV71 3C protein was expressed and mainly distributed in the cytoplasm at 6 h and 12 h post-infection (Fig. 4Bf and 4Bj), while majority of PCBP1 in the cytoplasm of EV71-infected cells (Fig. 4Bg and 4Bk) and co-localized with EV71 3C protein (Fig. 4Bh and 4Bl).

To further confirm the sub-cellular distribution of PCBP1 protein during EV71 infection, RD cells were infected with or without EV71. Whole cell lysate, cytoplasm extraction, and nucleus extraction were prepared from mock-infected or EV71-infected cells. PCBP1 protein was then detected by western blot analysis. Results showed that in whole cell lysates and in the cytoplasm, PCBP1 protein level was higher in EV71-infected cells than in mock-infected cells (Fig. 4C). However, in the nucleus, PCBP1 protein level was lower in EV71-infected cells than in mock-infected cells (Fig. 4C). The bar graph also indicated that in the cytoplasm, PCBP1 protein level was higher in EV71-infected cells than in mock-infected cells (Fig. 4C). These results demonstrated that PCBP1 was distributed in the nucleus and cytoplasm of mock-infected cells, but mainly distributed in the cytoplasm of EV71-infected cells, suggesting that EV71 infection affects the sub-cellular distribution of PCBP1 protein.

### PCBP1 is located in endoplasmic reticulum-derived membrane-associated complex during EV71 replication

**Figure 5:**
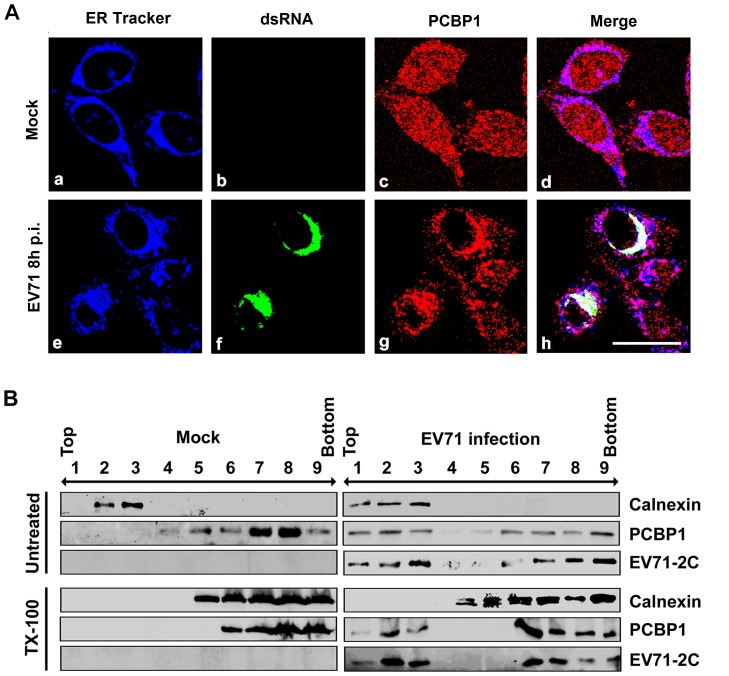
Localization of PCBP1 in ER-derived membrane-associated complex during EV71 replication. (A) RD cells were mock-infected or infected with EV71 at an MOI of 5 for 8 h. Cells were probed with rabbit anti-PCBP1 antibody and mouse anti-dsRNA antibody, stained with ER-Tracker, observed by confocal microscopy. PCBP1 (red), dsRNA (green), ER-Tracker (blue). Bar = 20 µm. (B) Membrane flotation analysis of location of PCBP1. RD cells were infected with or without EV71 at an MOI of 5 for 8 h. Cell lysates from mock-infected and infected cells treated with or without Triton X-100 were separated by sucrose gradient sedimentation. Each fraction was blotted with specific antibodies to the ER marker protein (calnexin, CNX), PCBP1, and EV71 2C, respectively. Fractions 1 to 3 represent the membrane fraction. Fractions 4 to 9 represent the cytosolic fraction.

Enteroviruses and flaviviruses can reorganize the host secretory trafficking between the endoplasmic reticulum-Golgi intermediate compartment (ERGIC) and the trans-Golgi network (Golgi/TGN) to generate membrane microenvironments suitable for viral replication [35]. To increase our understanding for the role of PCBP1 in EV71 replication, we evaluated the sub-cellular distributions of EV71 RNA, PCBP1, and endoplasmic reticulum (ER) membrane and the co-localization among these three components during EV71 infection. RD cells were infected with or without EV71, and then probed with ER-Tracker, anti-PCBP1 antibody, and anti-dsRNA antibody, and finally examined under confocal microscopy. In mock-infected cells, ER-Tracker was diffusely distributed in the cytoplasm (Fig. 5A a), EV71 RNA was not detected as expected (Fig. 5Ab), while PCBP1 was distributed throughout the nucleus and cytoplasm (Fig. 5Ac) and the ER-Tracker distribution was not affected by PCBP1 (Fig 5Ad). At 8 h post-infection, ER-Tracker was also diffusely distributed in the cytoplasm (Fig. 5Ae), EV71 RNA was distributed in the cytoplasm (Fig. 5Af), but PCBP1 was mainly distributed in the cytoplasm (Fig 5Ag) and co-localized with both ER-Tracker and EV71 RNA (Fig 5Ah), perhaps in the ER-derived membrane, where the virus replication was occurred. Taken together, these results suggested that in EV71-infected cells, PCBP1 and EV71 RNA co-localized in the ER-derived membrane during viral replication.

Most of RNA viruses replicate in the detergent-resistant membrane (DRM) fraction, such as HCV, PV, coxsachievirus B3 (CVB3), and rhinovirus (RV) [35], suggesting that the viral replication complex is associated with the lipid raft [29]. EV71 belongs to a member of enteroviruses, its nonstructural protein 2C contains a membrane binding region at residues 21–54 amino acid [7], viral RNA and 2C protein are associated with the DRM structure, and thus we then examined whether PCBP1 is associated with the DRM structure. RD cells were infected with or without EV71 for 8 h. Cell lysates were prepared from mock and infected cells, treated with or without Triton X-100, and separated by sucrose gradient sedimentation. Proteins from each fraction were detected by Western blot analysis. Results from membrane flotation analysis showed PCBP1 was located in the cytosolic fractions (Fig. 5B, fractions 4 to 9) in both mock-infected and EV71-infected cells. However, a proportion of PCBP1 and EV71 2C protein were associated with the membrane fractions in EV71-infected cells, but not in mock-infected cells (Fig. 5B, fractions 1 to 3). The membrane-associated PCBP1 and EV71 2C protein can not be removed by Triton X-100 treatment (Fig. 5B, fractions 1 to 3). As a control, an ER marker protein, calnexin (CNX) [36], was detected exclusively in the detergent-soluble membrane fractions (Fig. 5B, fractions 2 to 3) in both mock-infected and EV71-infected cells, which is characteristic of unmodified ER. These results suggested that a proportion of PCBP1 protein alone with EV71 2C protein were located in the DRM fractions in EV71-infected cells, indicating PCBP1 was specifically recruited to the EV71 replication complex.

### PCBP1 promotes EV71 viral protein expression and virus production

**Figure 6:**
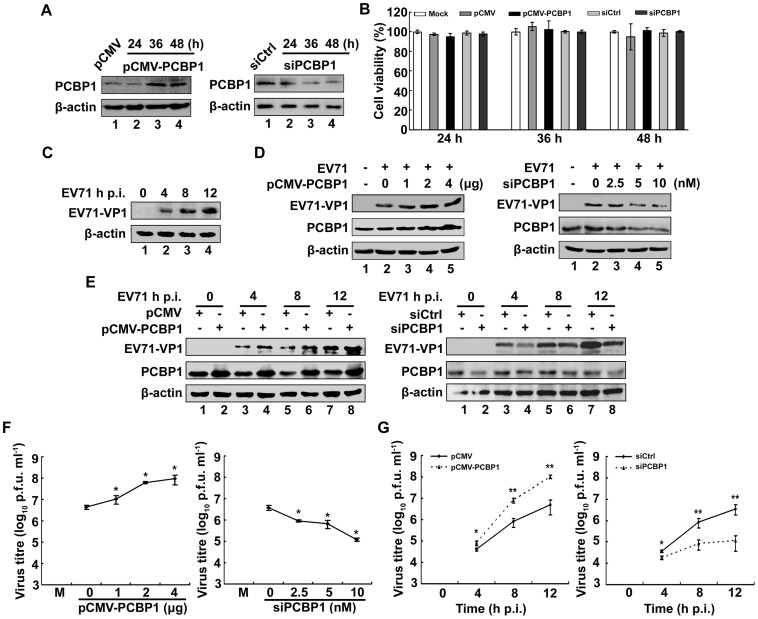
Analysis of PCBP1 in the regulation of EV71 viral protein expression and virus production. (A) RD cells were transfected with pCMV-PCBP1 (to over-express PCBP1), pCMV (a negative control of pCMV-PCBP1), siPCBP1 (to knock-down PCBP1) and siCtrl (a negative control of siPCBP1), respectively. At 24, 36, and 48 h post-transfection, cells extracts were prepared and proteins were detected by western blot using mouse anti-PCBP1 antibody or mouse anti-β-actin antibody. (B) RD cells were transfected with pCMV-PCBP1, pCMV, siPCBP1, and siCrtl, respectively. At 24, 36, and 48 h post-transfection, viability of transfected cells were determined by MTT assay. The viability of mock cells was normalized to 100%. Data are from three independent experiments with similar results. (C) RD cells were infected with or without EV71 at an MOI of 5 for 4, 8, and 12 h. Mock-infected and EV71-infected cells were lysed and proteins were detected by western blot analysis using antibodies to EV71 VP1 protein or β-actin protein. (D) RD cells were transfected with pCMV-PCBP1 at different concentrations (0, 1, 2, and 4 µg) or with pCMV at different concentrations (4, 3, 2, and 0 µg), siPCBP1 at different concentrations (0, 2.5, 5, 10 nM) or with siCtrl at different concentrations (10, 7.5, 5, 0 nM), respectively, for 36 h, and then infected with or without EV71 at an MOI of 5 for 12 h. Mock-infected and EV71-infected cells were lysed and proteins were detected by western blot analysis using antibodies to EV71 VP1 protein, PCBP1 or β-actin protein. (E) RD cells were transfected with pCMV-PCBP1, pCMV, siPCBP1, and siCtrl, respectively, for 36 h, and then infected with EV71 at an MOI of 5 for 4, 8, and 12 h. EV71-infected cells were lysed and proteins were detected by western blot analysis using antibodies to EV71 VP1 protein, PCBP1 or β-actin protein. (F) Cell culture supernatants from (D) were prepared and subjected to plaque assays and viral infectivity titers of culture supernatants (plaque forming units per ml, PFU/ml) on EV71-infected RD cells were determined. (G) Cell culture supernatants from (E) were collected and viral infectivity titers in supernatants were determined. Viral infectivity titers are expressed as means ± S.E. (n = 3) of three independent experiments (**, *p*<0.01; *, *p*<0.05).

Since we revealed that PCBP1 can bind to EV71 5′UTR RNA and is located in viral replication complex, the effect of PCBP1 on EV71 replication was then investigated. RD cells were transfected with pCMV-PCBP1 or pCMV, or treated with siPCBP1 or siCrtl, respectively. Western blot results showed that PCBP1 protein was detected in cells transfected with pCMV and the level of PCBP1 was increased in cells transfected with pCMV-PCBP1 at 36 h and 48 h post-transfection (Fig. 6A, left panel). PCBP1 protein was also detected in cells transfected with siCrtl, but the level of PCBP1 was decreased in cells transfected with siPCBP1 at 36 h and 48 h post-transfection (Fig. 6A, right panel). Results from MTT assay showed that cell viability was not affected by pCMV-PCBP1, pCMV, siPCBP1, and siCtrl at 24, 36, and 48 h post-transfection (Fig. 6B). These results indicated that pCMV-PCBP1 and siPCBP1 are effective and that PCBP1 and siPCBP1 have no effect on cell viability. RD cells were infected with or without EV71 for different times as indicated. Western blot analysis showed that EV71 VP1 protein was not detected in mock-infected cells, but expressed in EV71-infected cells in a time-dependent fashion as expected (Fig. 6C), indicating that EV71 is replicated in the cells.

RD cells were then transfected with pCMV-PCBP1 or pCMV, siPCBP1 or siCtrl at different concentrations, as indicated, and then infected with EV71. Western blot results revealed that EV71 VP1 protein was not detected in mock-infected cells, but detected in EV71-infected cells as expected. In addition, the level of VP1 protein was gradually increased as the concentration of PCBP1 increased (Fig. 6D, left panel), but gradually reduced as the concentration of siPCBP1 increased (Fig. 6D, right panel), revealing that PCBP1 activates EV71 viral protein expression. To further confirm the roles of PCBP1 in regulating dynamic expression of EV71 viral protein, RD cells were transfected with pCMV-PCBP1 or pCMV, siPCBP1 or siCtrl, and then infected with EV71 for different times, as indicated. Results showed that EV71 VP1 protein was not detected in mock-infected cells (Fig. 6E, lanes 1 and 2), expressed in EV71-infected cells (Fig. 6E, lanes 3-8). In addition, the level of EV71 VP1 protein was increased as the infection time increased (Fig. 6E, lanes 3, 5, and 7), but further enhanced by over-expression of PCBP1 (Fig. 6E, left panel, lanes 4, 6, and 8) and reduced by knocked-down of PCBP1 (Fig. 6E, right panel, lanes 4, 6, and 8) in a time-dependent manner. Taken together, our results demonstrated that PCBP1 enhances EV71 viral protein expression.

To evaluate the effect of PCBP1 on EV71 virus production, RD cells were transfected with different concentrations of pCMV-PCBP1, pCMV, siPCBP1, or siCtrl, respectively, and then infected with EV71 for 12 h. Cell culture supernatants were prepared and subjected to plaque assays and the titers of viral infectivity of EV71 were determined in infected-RD cells. Results showed that virus production and the levels of viral titers in EV71-infected cells were enhanced by over-expression of PCBP1 (Fig. 6F, left panel), but reduced by knock-down of PCBP1 (Fig. 6F, right panel) in a dose-dependent manner, suggesting that PCBP1 activates EV71 virus production. To further confirm the role of PCBP1 in the regulation of EV71 production, RD cells were transfected with pCMV-PCBP1, pCMV, siPCBP1, or siCtrl and then infected with EV71 for different times. Results from plaque assay indicated that the level of EV71 viral titer was activated by over-expression of PCBP1 (Fig. 6G, left panel) and reduced by know-down of PCBP1 (Fig. 6G, right panel) in a time-dependent manner. Results revealed PCBP1 facilitates EV71 viral replication.

## Discussion

Positive-strand RNA viruses initiate translation upon entry of the genomic RNA to host cells and start to duplicate the RNA from the 3′ end resulting in generation of a complementary negative-strand RNA, which is transcribed into a nascent positive genomic RNA. This replication cycle can produce thousands of copies of the genome within a few hours [37]. Thus, viral genomic RNA requires efficient machinery for replication and translation, in which viral and host cell proteins associated with viral genomic RNA are known to play important roles. Viral RNA functional regions that direct RNA translation and replication are usually located at the 5′ or 3′ ends. The 5′UTR of viral RNA, such as HCV [38], [39] and PV [40], [41], plays roles in both translation and replication of the viral genome in concert with cellular proteins, including PCBP1 and PCBP2 [33].

PCBP1 binds to the 3′ non-coding region (3′ NCR) of cellular mRNAs and plays a role in the stabilization or translational control of mRNAs [16], [17], [18] and also interacts with the 5′UTR of poliovirus RNA to facilitate the viral replication [19], [20]. PCBP2, a homolog of PCBP1, regulates cap-independent translation of poliovirus [42] and interacts with the 5′UTR of HCV RNA to direct the viral RNA replication [29]. However, the role of PCBP1 in the regulation of EV71 replication, especially the sub-cellular location of PCBP1 during viral replication, has not been reported and the mechanism involved in such regulation is unknown.

In this study, the binding ability of PCBP1 to the 5′UTR of EV71 RNA was first evaluated. By using biotinylated RNA/protein binding assay, we revealed that cellular PCBP1 protein can specifically bind to the 5′UTR of EV71 RNA in RD, HeLa, and SK-N-SH cells infected with the virus. The 5′UTR of EV71 RNA comprises 745 nt and forms a secondary structure. We predicted that it contains six stem-loops (I–VI) with three functional regions (cloverleaf, IRES, and linker) and demonstrated that stem-loop I (nt 1–100) and IV (nt 230–450) in EV71 5′UTR are required for the binding of PCBP1 to the viral RNA.

PCBP1 contains three RNA-binding domains, KH1, KH2, and KH3. KH1 and KH3 bind to poly(C) homopolymers and KH1 acts as a dominant-negative mutant to inhibit translation of poliovirus [15], [43]. In an effort to determine the functions of PCBP1 KH domains, we revealed that the KH1 domain of PCBP1 is required for the binding of PCBP1 to EV71 5′UTR. Thus, we proposed that KH1 domain of PCBP1 specifically binds to stem-loop I and IV of EV71 5′UTR, which may play an important biological function in viral replication.

PCBP proteins are frequently distributed throughout the cells, including the nucleus and cytoplasm [34]. Many positive-strand RNA viruses, poliovirus, HCV, and enterovirus, replicate and translate in the cytoplasm of host cells [35]. However, the sub-cellular distribution of PCBP1 during EV71 infection was unclear. Cells infected with viruses undergo a dramatic remodeling of intracellular membranes, and RNA replication takes place on the cytosolic leaflet of remodeled membranes [27], [44], [45]. Replication membranes for positive-strand RNA viruses appear to originate from the endoplasmic reticulum (ER) [46], [47]. To increase our understanding for the role of PCBP1 during EV71 replication, we investigated the sub-cellular distributions of PCBP1 protein, and EV71 RNA during viral infection. Our results revealed that PCBP1 is distributed throughout the nucleus and cytoplasm in mock-infected cells. In EV71-infected cells, through interacting and co-localizing with the viral RNA, PCBP1 was mainly distributed in the cytoplasm where EV71 replication (indicated as dsRNA and EV71 3C as well) was mainly occurred.

ER membrane is distributed in the cytoplasm of mock-infected cells, while the viral dsRNA, ER membrane and PCBP1 were distributed in the cytoplasm of EV71-infected cells in a co-localized fashion. The sub-cellular distribution of ER membrane was not affected by PCBP1 in mock-infected cells, however, PCBP1, and EV71 RNA were focused and mainly distributed in the cytoplasm and the three components, EV71 dsRNA, ER membrane, and PCBP1 protein, were co-localized in the cells, especially in the ER-derived membrane complex or DRM. These results demonstrated that the cellular factors and the viral RNA were co-localized in EV71-infected cells, suggesting that PCBP1 interacts with EV71 RNA in the ER-derived membrane complex.

The role of PCBP1 in EV71 viral protein expression was then investigated. We revealed that over-expression of PCBP1 resulted in the stimulation of EV71 VP1 protein expression and knock-down of PCBP1 resulted in inhibition of EV71 VP1 protein expression, indicating PCBP1 plays a stimulatory role in regulation of EV71 protein expression. The effect of PCBP1 on the production of EV71 virus was also determined in this study. We showed that in EV71-infected cells, over-expression of PCBP1 enhances the viral titers and knocked-down of PCBP1 reduces the viral titers, indicating PCBP1 plays a stimulatory role in EV71 viral production. Thus, PCBP1 can be viewed as an important cellular factor in EV71 replication.

It's known that many RNA virus 5′UTR is fundamentally important for virus replication by interacting with cellular proteins, including viral RNA translation and synthesis [40], [41]. Sine we revealed that PCBP1 binds to stem-loop I and IV of EV71 5′UTR, and it has been previously demonstrated that stem-loop I forms a cloverleaf structure necessary for poliovirus negative-strand synthesis [14] and stem-loop IV is located in the IRES region of poliovirus and coxsackievirus 5′UTR required for IRES-mediated translation of the viral RNA [42], [48], our results more likely indicated that the interaction between PCBP1 and EV71 5′UTR resulted in facilitating viral replication, which is in agreement with previous reports that PCBP2 is required for the activation of poliovirus IRES, and thus for the translation of the viral RNA [49]. During poliovirus replication, PCBP2 mediates the switch from translation to RNA replication. It is possible that the viral replication cycle is a dynamic process with both activities occurring simultaneously [43]. In many positive-strand RNA viruses, various cellular proteins required for viral replication affect virus production based on the characteristics of the virus itself, and the direct and indirect impacts of cellular proteins on viral replication during its life cycle [50], [51]. Several cellular proteins, hnRNP K, FUBP1, and FUBP2, are redistributed to the translation of EV71 genome [9], [11], [12], PCBP1 is also redistributed in cytoplasm to act as a positive factor during EV71 replication.

Overall, our research reveals one cellular factor, PCBP1, as being an important regulator of EV71 replication, has affinity for viral 5′UTR and is located in viral membrane-associated complex to promoter viral replication. These findings not only improve our understanding of cellular factors involved in EV71 replication, but also provide a new strategy to potential anti-viral drugs by inhibiting EV71 replication.
